# Immature teratoma of the posterior cranial fossa in a 4-month-old infant: A case report

**DOI:** 10.3892/ol.2013.1325

**Published:** 2013-04-29

**Authors:** JINXI GAO, ZHAOCONG ZHENG

**Affiliations:** Department of Neurosurgery, Fuzhou General Hospital of Nanjing Military Command, Fuzhou, Fujian 350025, P.R. China

**Keywords:** teratoma, posterior cranial fossa, infant, hydrocephalus, chemotherapy

## Abstract

The present study analyzed a case of immature teratoma in the posterior cranial fossa of an infant and compared the clinical data with the associated literature. Ventricular drainage was initially performed upon the patient’s admission to the hospital. Following adequate pre-operative preparations, the tumor in the posterior cranial fossa was resected on the third day. No significant neurological function deficiency was observed following the surgery and no recurrence was noted within an 18-month follow-up period. In such cases, treatment should be conducted in a stepwise manner, with the hydrocephalus relieved first, followed by complete tumor resection subsequent to full preparation. Post-operative chemotherapy was not performed by conventional means as the infant was too weak, therefore, periodic reviews and long-term follow-up were required.

## Introduction

Teratomas are ectopic tumors containing multiple tissues from more than one mesoderm. Encephalic teratomas are rare and account for 2 to 5% of infant teratomas and 0.3 to 0.6% of all ventricular tumors. Teratomas may occur at any age, but with a higher incidence rate in younger patients (1). In addition, the incidence rate in males is marginally higher than that in females. Encephalic teratomas always occur in the midline, where they are typically detected in the pineal and suprasellar regions and rarely in the posterior cranial fossa. Obstructive hydrocephalus is a common complication of teratomas (2). This disease is difficult to diagnose due to its rarity. Furthermore, encephalic teratomas are occasionally misdiagnosed as highly malgnant tumors. For this reason, the family members of patients reject treatment and infant patients may lose the chance to be cured of obstructive hydrocephalus. An accurate pre-operative diagnosis is important and patients are usually in an extremely dangerous condition when they are admitted to hospital. As such, effective treatment strategies create conditions that promote the post-operative recovery of patients and lay the foundation of the desired long-term prognosis. In the present study, an infant patient with a large immature teratoma in the posterior cranial fossa, accompanied by obstructive hydrocephalus, was admitted to the Department of Neurosurgery at Fuzhou General Hospital of Nanjing Military Command (Fuzhou, China) in January 2011. The treatment exhibited a positive effect on the patient. Strategies identified in the associated literature were used to conduct the analyses. Written informed consent was obtained from the patient’s family.

## Case report

**Figure 1.**
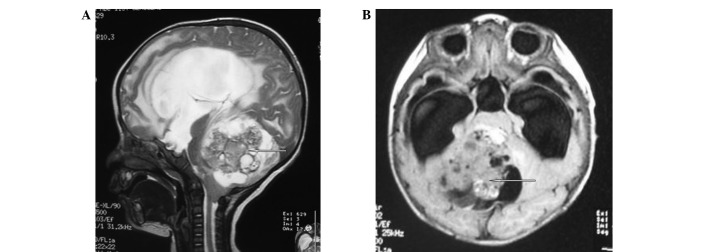
Posterior cranial fossa teratoma (arrowheads) accompanied by hydrocephalus. (A) Sagittal and (B) axial magnetic resonance imaging (MRI) scans.

A male infant aged 4 months and 9 days was admitted to Fuzhou General Hospital of Nanjing Military Command with disease symptoms, including binocular convergence, a downward gaze for >10 days and sudden seizures for one day. The infant was vaccinated according to the recommended immunization schedule and no clear abnormalities were observed. The size of the infant’s head was normal for his age. The infant was born mature to a 34-year-old G3P3 mother, without any history of exposure to X-rays and toxic substances. No abnormalities were observed during the routine antenatal care. The infant weighed 3.5 kg at birth and did not have a history of asphyxia rescue. The patient’s Apgar score was 9. Breast-feeding was conducted after birth. A special examination of the infant revealed the following: a 5×5 cm bregma that was plump and bulging to a height of ∼3 mm, and a head size of 49 cm. Macewen’s sign was observed upon skull percussion. Additionally, the infant was in a moderate coma. The setting-sun sign was observed in the patient’s eyeball, while the bilateral pupil of the infant faced downwards, had a diameter of 1.5 mm and lagged in response to direct and indirect light. Muscular tension in the four limbs of the infant patient was reduced and limbs bent upon pain stimulation. Head magnetic resonance imaging (MRI) scans of the infant showed a quasi-circular long signal in the cerebellar vermis, which exhibited a marginally high signal in the water-suppression image. The lesion was ∼5.8×5.7×6.0 cm in size and had a clear boundary. The signal of the lesional substance was uneven. Small, circular long T1 and T2 signals and short, irregular T1 and T2 signals were also observed. The brain stem was compressed and deviated forward. The fourth cerebral ventricle had become narrowed due to compression. The third cerebral and two lateral ventricles were expanded. A small patch of long T1 and T2 signals was observed around the lateral ventricles, which showed a high signal in the water-suppression image. The fissures and sulci on each side were not broadened and the middle structures of the brain remained at the midline (Fig. 1). The clinical diagnosis was as follows: i) cerebellar-space-occupying lesion, local cystic lesion and bleeding; and ii) obstructive hydrocephalus.

**Figure 2.**
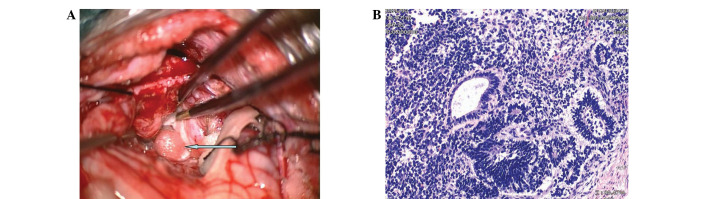
View under the microscope during (A) surgery and (B) a pathological section examination. (H&E; magnification, 40X). The teratomas were composed of tissues derived from the three germ layers, containing epithelial tissue components, immature areas and foci of calcification (arrowheads).

**Figure 3.**
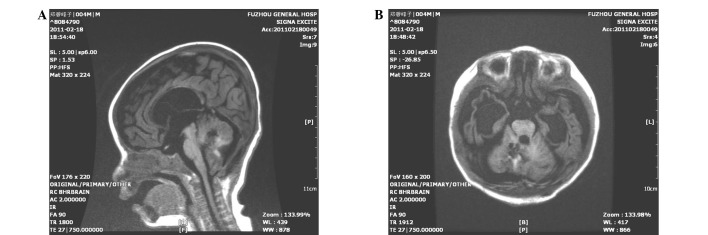
Post-operative review showing the disappearance of the tumor and the subsidance of the hydrocephalus. (A) Sagittal and (B) axial magnetic resonance imaging (MRI).

Ventricular drainage was performed through a puncture in the anterior fontanelle as an emergency treatment once the patient had been admitted to the hospital. The patient’s consciousness gradually returned following the surgery. Tension in the anterior fontanelle was reduced. Thus, a fluid infusion was performed to stabilize the patient’s condition. After full preparations had been made, including fluid infusion, blood preparation and the use of intraoperative pathology, the cerebellar vermis tumor was resected from the posterior midline of the patient under general anesthesia on the third day. The tumor was located in front of the cerebellar vermis during the surgery and was lobulated with an irregular shape and uneven texture. Certain sections of the tumor were soft, whereas others were tough. Cystic changes were observed in several places. A large amount of the cyst fluid was yellow, whereas the remainder was white and dirty. In addition, several light-red small circular nodes, with a diameter of ∼3 mm and rough surfaces, were observed (Fig. 2A). Calcified points and sporadic white hair were also observed. The tumor had a full membranous envelope with an average blood supply and a clear dividing line. The tumor pressed on the cerebellum and the bottom of the four ventricles, with unclear peripheral adhesions. The tumor was fully resected under the guidance of a microscope. The pathological result was a Grade II immature teratoma (Fig. 2B). The post-operative recovery was good, with the patient showing an improved mental response. MRI scans revealed that the tumor had been completely removed (Fig. 3). Moreover, the hydrocephalus was also significantly improved. The infant was discharged from the hospital and follow-ups were performed for 18 months. No recurrence of the neoplasm was noted. At present, the patient is experiencing normal growth and development.

## Discussion

Intracranial teratomas exhibit different clinical features according to the growth zones. In the majority of infant patients, the main clinical feature of these tumors is intracranial hypertension. Intracranial teratomas are commonly accompanied by symptoms that include obstructive hydrocephalus, which results in headaches, vomiting, papilledema, outreach paralysis and progressive increases in head size. A small number of infant patients also experience epileptic attacks, even when they are in a coma, which may be associated with the intracranial hypertension and brainstem compression caused by the tumor. Infant patients with posterior cranial fossa teratomas do not exhibit dystaxia since their motor function is not yet fully developed. The reason that a medical consultation is required for these infant patients is typically the presentation of hydrocephalus or epilepsy (3). MRI has advantages in characterizing the shape, texture, outline, composition and original position of the tumor, as well as its association with surrounding structures, particularly in enhanced scanning. MRI is able to clearly identify the original position of the tumor and its invasiveness and subsequently provide guidance in surgery (4).

In the present study, the patient was admitted to the Fuzhou General Hospital of Nanjing Military Command due to binocular cohesion and a downward gaze for >10 days, and sudden hyperspasmia for one day. These symptoms are features of hydrocephalus and epilepsy. The patient did not undergo immediate tumor resection since he was weak and the hydrocephalus was serious. Therefore, the hydrocephalus was resolved first. Tumor resection was then performed from the posterior midline under general anesthesia on the third day after full pre-operative preparations had been made. A good therapeutic effect was subsequently achieved. A lateral ventricle puncture through the bregma was a suitable choice since the infant bregma was not yet closed. This surgery is simple and easy and is able to rapidly relieve severe intracranial hypertension caused by hydrocephalus, thereby stabilizing the vital signs of infant patients. This allows doctors more time to perfect the pre-operative preparations and improve the safety of the tumor resection. However, a ventricle puncture may cause transtentorial herniation when posterior cranial fossa occupation is accompanied by obstructive hydrocephalus. Such a case would require more attention during treatment. Resection is the main method for treating posterior cranial fossa teratomas. In solving the hydrocephalus, doctors should attempt to remove as much of the tumor as possible. The key aspect of treating a teratoma is opening the circulation passage of the cerebrospinal fluid. This type of tumor has a complete membranous envelope with a clear boundary and a low blood supply. Therefore, as much of the tumor as possible should be removed. Since tumors are often located within important brain structures, the surgery should be performed carefully to avoid damaging other organs. Teratoma surgeries should also be conducted carefully for sections of teratomas that exhibit cystic changes, in order to prevent cyst fluid from flowing into the resorption cavity of the arachnoid space, thereby preventing post-operative pyrexia.

Platinum-based chemotherapy is the main post-operative chemotherapy for immature teratomas (5). The most common chemotherapy regimen consists of cis-platinum, bleocin and etoposide or cyclophosphamide, combined with taxol. The difference in the sensitivity to chemotherapy among teratomas is high due to the various compositions of the tumor tissue (6). Overall, teratomas are not highly sensitive to post-operative chemotherapy. In addition, chemotherapeutics may have certain toxic effects, such as digestive tract symptoms, bone marrow arrest, renal toxicity, auditory nerve damage, pneumoedema and pulmonary fibrosis. Infant patients are weak and have difficulty enduring these complications. Thus, post-operative chemotherapy is not recommended as a routine treatment for infant patients (7). The infant in the present study was only four months old and was weak, therefore, chemotherapy was not administered.

The prognosis for an immature teratoma is associated with tumor differentiation. Highly-differentiated tumors have a better prognosis, while those that are mainly composed of undifferentiated embryonal tissue have a worse prognosis. Ogawa *et al* (8) reported that the 10-year survival rate of immature teratoma was up to 70%. Full tumor resection may suspend the development of the disease to a certain extent, although infants who survive surgery may have varying levels of developmental retardation (9). Teratomas may be detected in various locations and generate germ cell tumors with different histological types. Therefore, periodic checkups and a long-term follow-up should be performed, even if the computed tomography (CT) and MRI results indicate a full resection. The early recurrence of a teratoma may be identified by monitoring the cerebrospinal fluid and serum tumor markers. Therefore, periodic head MRI scans are essential between 6 months and 3 years post-surgery (10). Long-term recurring tumors should not be blindly diagnosed as the recurrence of a teratoma and the possibility of the patient having other malignant germ cell tumors should also be considered (11). In the present study, follow-ups were conducted for 18 months. No recurrence or abnormal development were noted. However, attention continues to be given to the prognosis of this infant patient due to the short follow-up duration.

In conclusion, infant patients with immature teratomas in the posterior cranial fossa are brought to see doctors mainly due to the symptoms of hydrocephalus and epilepsy. Ventricular drainage through an anterior fontanelle puncture is an effective measure for treating patients with severe hydrocephalus. Immature teratomas have the potential to change into other malignant tumors, thus, a long-term follow-up should be conducted for patients even if a full resection has been performed.
